# Supplementary material for: Contrasting factors associated with COVID-19-related ICU admission and death outcomes in hospitalised patients by means of Shapley values
Source: PLoS Comput Biol. 2021 Jun 23;17(6):e1009121. doi: 10.1371/journal.pcbi.1009121 (PMC8259985; doi:10.1371/journal.pcbi.1009121)
Supplement: S1 Text — Supporting Information, including: Figs A, B, C, D, E, and F, and Table A. (PDF) [file pcbi.1009121.s001.pdf]

Supporting Information to “Contrasting factors associated with COVID-19-related ICU admission and death outcomes in hospitalised patients by means of Shapley values”

Massimo Cavallaro[1,2*], Haseeb Moiz[3], Matt J. Keeling[1,2], Noel D. McCarthy[1,3,4*]

[1]The Zeeman Institute for Systems Biology & Infectious Disease Epidemiology Research, University of Warwick, Coventry, United Kingdom.

[2]School of Life Sciences and Mathematics Institute, University of Warwick, Coventry, United Kingdom.

[3]Warwick Medical School, University of Warwick, Coventry, United Kingdom.

[4]Institute of Population Health, Trinity College Dublin, University of Dublin, Dublin, Ireland.

*To whom correspondence should be addressed: M.Cavallaro@warwick.ac.uk (MC) and Noel.Mccarthy@tcd.ie (NDM)

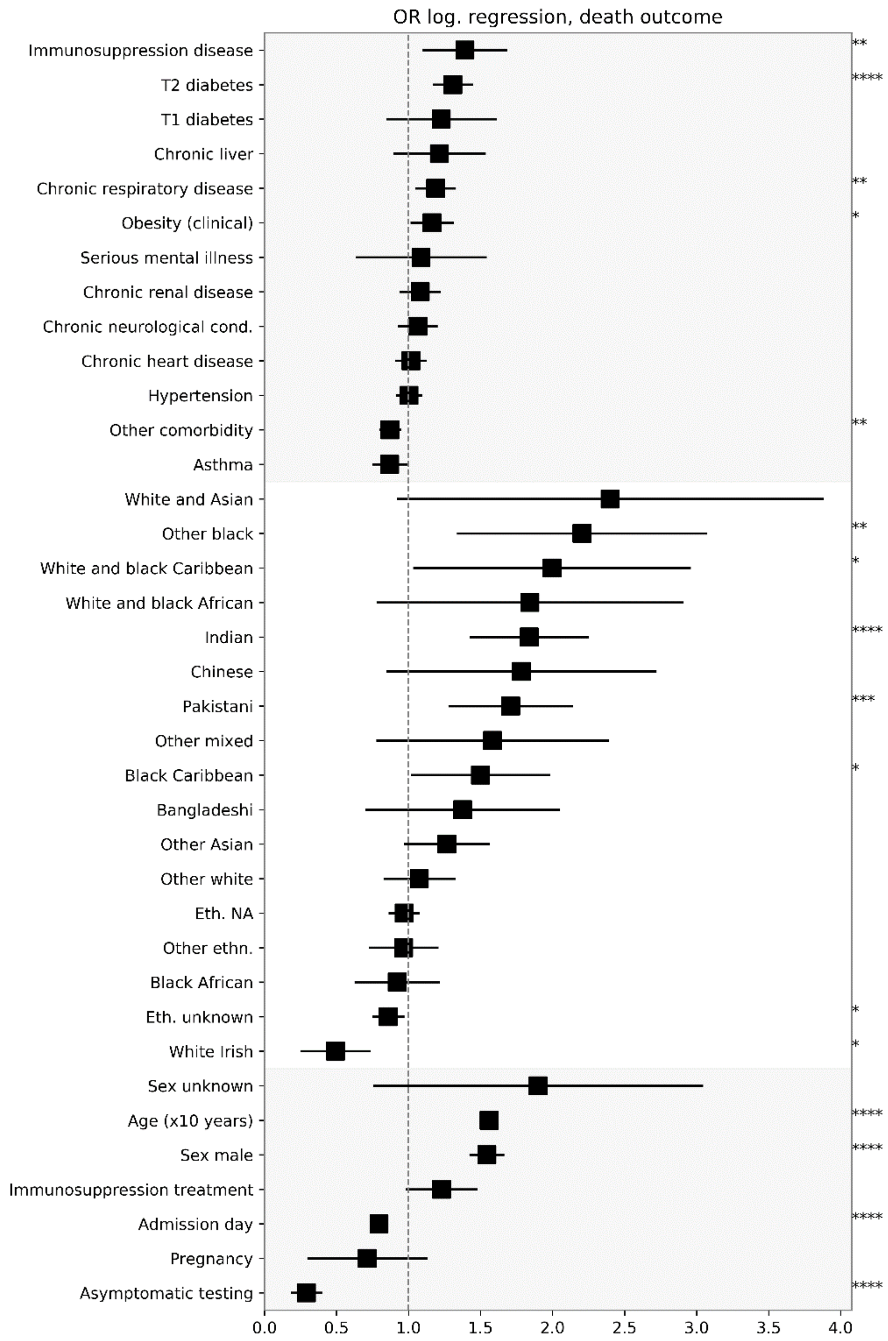

Fig A. Odd ratios (ORs) for death outcomes from Table 3. Features are grouped in comorbidities, ethnicities, and others (top to bottom). Significance star codes are \`*\` P ≤ 0.05, \`**\` P ≤ 0.01, \`***\` P ≤ 0.001, \`****\` P ≤ 0.0001.

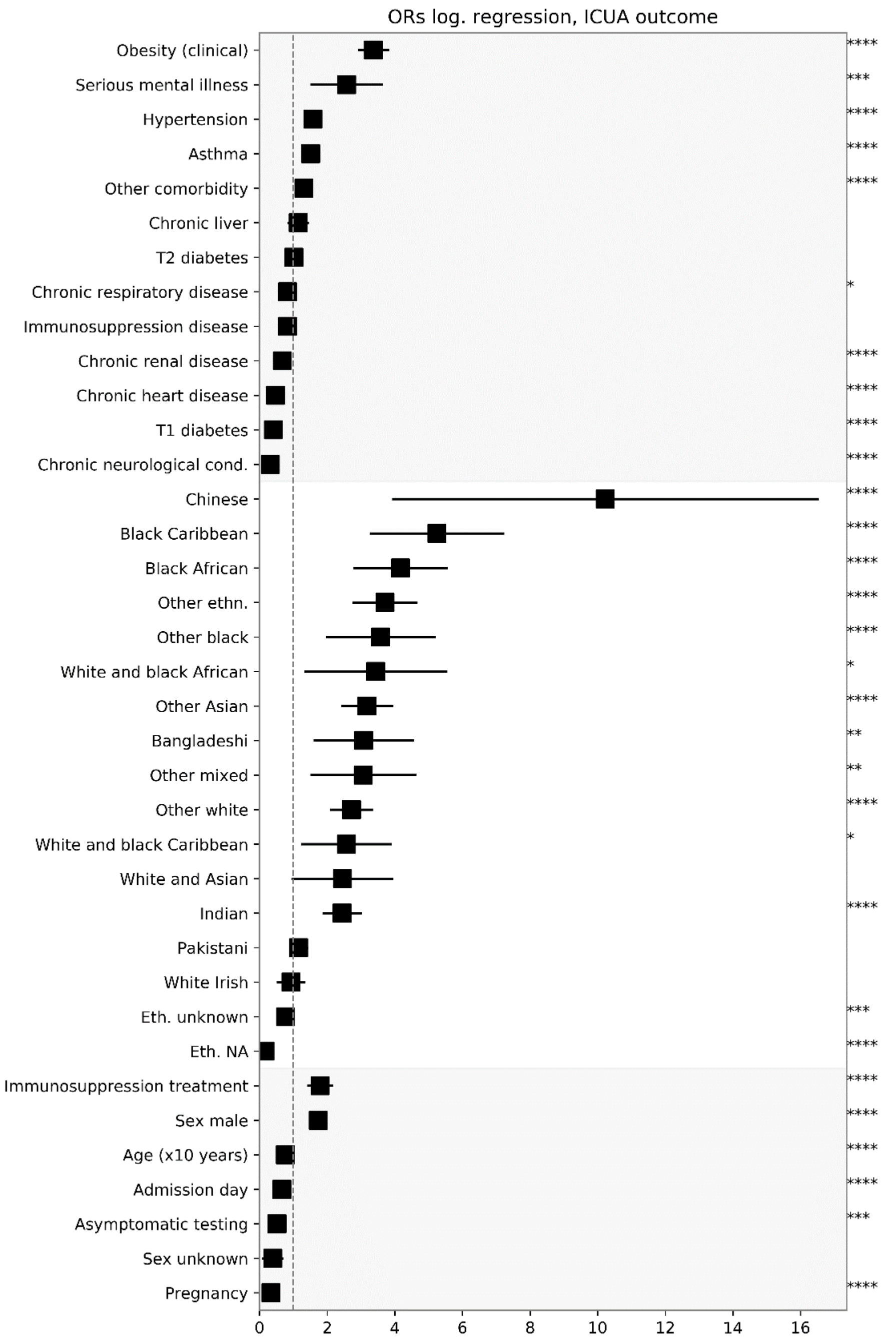

Fig B. Odd ratios (ORs) for ICUA outcome from Table 3. Features are grouped in comorbidities, ethnicities, and others (top to bottom). Stars are codes for significance (* P ≤ 0.05, **P ≤ 0.01, ***P ≤ 0.001,**** P ≤ 0.0001).

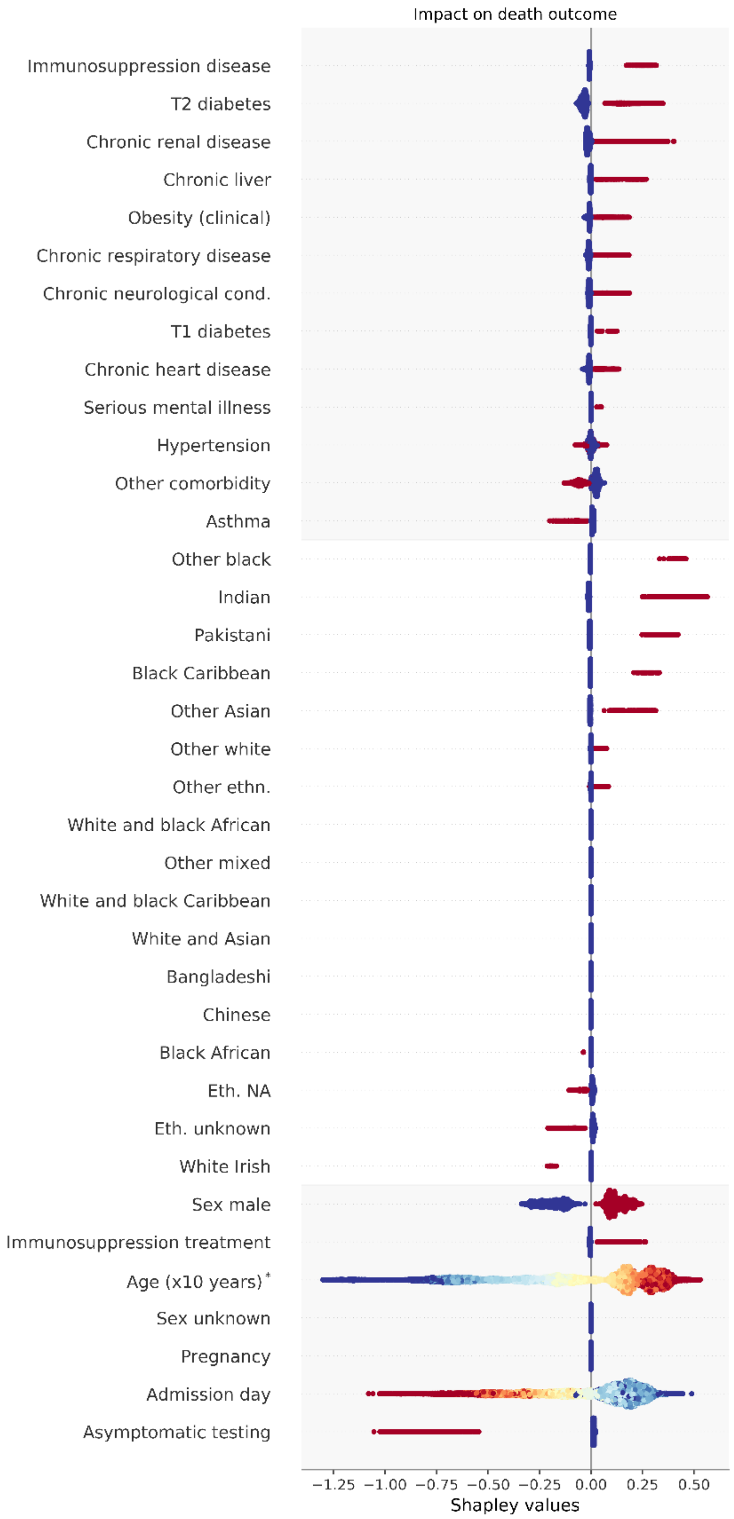

Fig C. Summary plot of Shapley values for impact on death outcome. For each potential risk factor, the Shapley values of each patient are represented as a swarm plot distribution. Colours from red to blue indicate the value of the underlying variable (in binary variables, red color means feature is present, blue otherwise; in age feature, red to blue shades correspond to old to young ages; in admission day, red to blue shades correspond to early to late dates). Features are grouped in comorbidities, ethnicities, and others. Within each group, the factors are ranked according to the importance score *Imp* (see Table 3 in main text); in other words, upper in the list are the conditions most likely to be associated with

the worst outcome. Immunosuppression by disease, type-2 diabetes mellitus, being male, and chronic liver, renal, respiratory and neurological conditions consistently appear to have positive impact to death outcome for all patients. Asthma was found to have negative impact on death for all patients. *Shapley values for age (x10 years) are scaled by a factor 0.5 to fit the plot range.

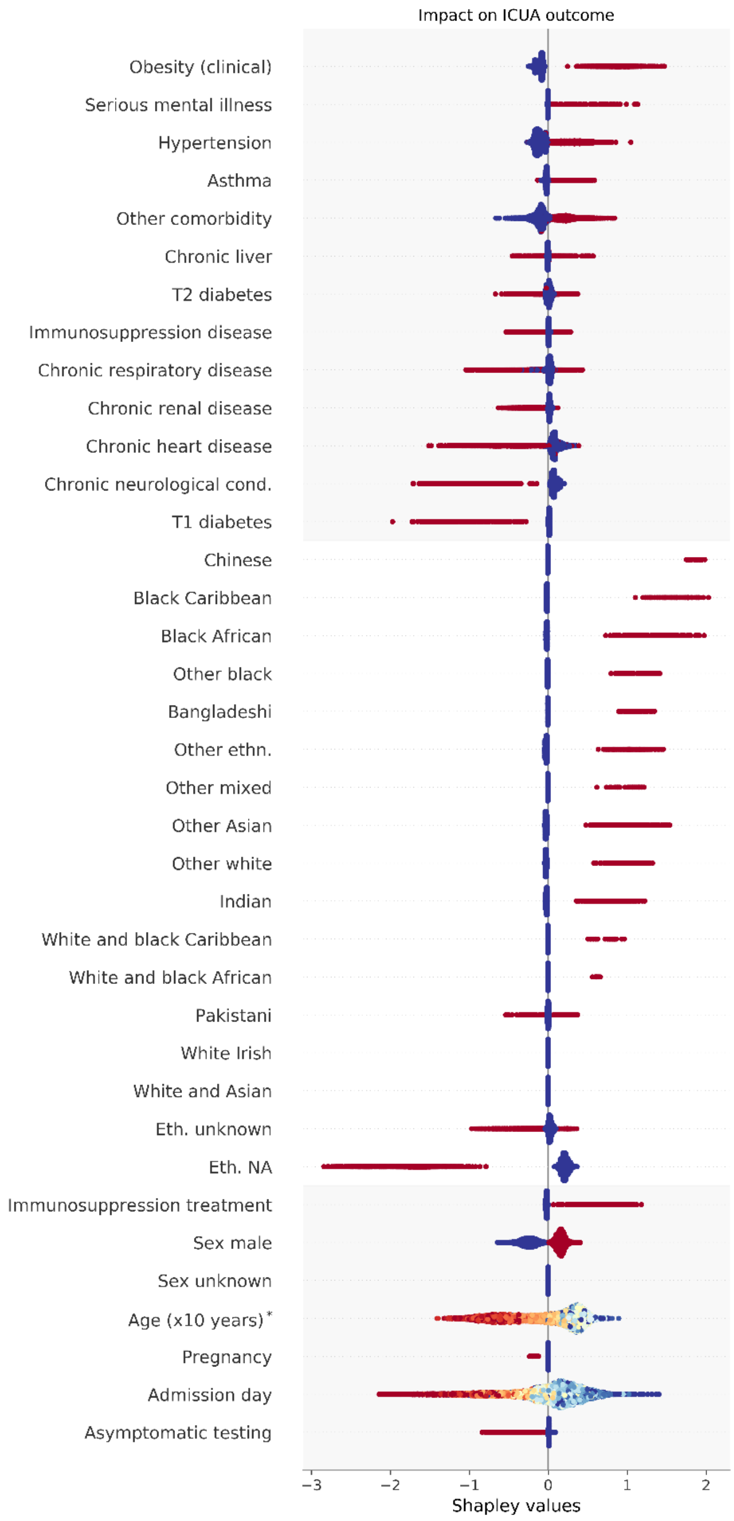

Fig D. Summary plot of Shapley values for impact on ICUA outcome. Colours and keys as in Fig C. The presence of obesity, hypertension, immunosuppression treatment, and "other comorbidity" were clear indicators of ICUA outcome for all patients. *Shapley values for age (x10 years) are scaled according to Fig C.

Table A. Estimated odd ratios (ORs) and variance inflation factors (VIFs) from logistic regression, importance (Imp) scores of death, and Benjamini-Hochberg (BH) significance test results adjusted for intensive-care unit admission (ICUA).

	OR	2.50%	97.50%	Pr(>|z|)	VIF	Imp	BH test
ICUA	2.25	2.04	2.48	0.00	1.48	0.39	TRUE
**Comorbidities:**							
Immunosuppr. disease	1.45	1.14	1.84	0.00	1.05	0.22	TRUE
T1 diabetes	1.44	0.99	2.08	0.05	1.02	0.12	FALSE
T2 diabetes	1.32	1.18	1.48	0.00	1.19	0.16	TRUE
Chronic neurological cond.	1.24	1.08	1.43	0.00	1.10	0.15	TRUE
Chronic liver	1.24	0.91	1.67	0.17	1.02	0.21	FALSE
Chronic respiratory disease	1.23	1.08	1.40	0.00	1.10	0.12	TRUE
Chronic renal disease	1.13	0.98	1.31	0.09	1.15	0.15	FALSE
Chronic heart disease	1.11	0.99	1.25	0.06	1.26	0.05	FALSE
Serious mental illness	1.03	0.60	1.71	0.92	1.02	0.00	FALSE
Obesity (clinical)	0.99	0.87	1.14	0.94	1.15	0.00	FALSE
Hypertension	0.94	0.85	1.03	0.19	1.27	-0.03	FALSE
Other comorbidity	0.84	0.77	0.92	0.00	1.17	-0.08	TRUE
Asthma	0.82	0.71	0.96	0.01	1.05	-0.16	TRUE
**Ethnicities:**							
White and Asian	2.08	0.79	5.44	0.13	1.01	0.15	FALSE
Other black	1.84	1.11	3.05	0.02	1.02	0.26	TRUE
White and black Caribbean	1.72	0.89	3.31	0.10	1.01	0.12	FALSE
Pakistani	1.69	1.26	2.27	0.00	1.04	0.33	TRUE
White and black African	1.59	0.66	3.62	0.28	1.01	0.11	FALSE
Indian	1.59	1.22	2.06	0.00	1.05	0.29	TRUE
Other mixed	1.36	0.66	2.66	0.38	1.01	0.04	FALSE
Chinese	1.31	0.61	2.76	0.48	1.01	0.00	FALSE
Eth. NA	1.23	1.09	1.39	0.00	1.19	0.09	TRUE
Black Caribbean	1.19	0.80	1.74	0.39	1.02	0.04	FALSE
Bangladeshi	1.18	0.60	2.23	0.63	1.01	0.05	FALSE
Other Asian	1.05	0.80	1.37	0.74	1.05	0.04	FALSE
Other white	0.92	0.70	1.20	0.54	1.03	0.01	FALSE
Eth. unknown	0.90	0.78	1.04	0.15	1.07	-0.07	FALSE
Other ethn.	0.81	0.60	1.07	0.15	1.04	-0.15	FALSE
Black African	0.74	0.50	1.07	0.12	1.03	-0.18	FALSE
White Irish	0.51	0.26	0.95	0.04	1.00	-0.39	FALSE
**Other:**							
Sex unknown	2.09	0.82	5.27	0.12	1.01	0.39	FALSE
Age (x10 years)	1.67	1.62	1.73	0.00	1.44	0.01	TRUE
Sex male	1.45	1.34	1.58	0.00	1.05	0.11	TRUE
Immunosuppr. treatment	1.14	0.91	1.43	0.24	1.06	0.09	FALSE
Admission day	0.84	0.80	0.87	0.00	1.09	-0.20	TRUE
Pregnancy	0.83	0.34	1.81	0.66	1.01	0.00	FALSE
Asymptomatic testing	0.31	0.19	0.47	0.00	1.04	-0.73	TRUE

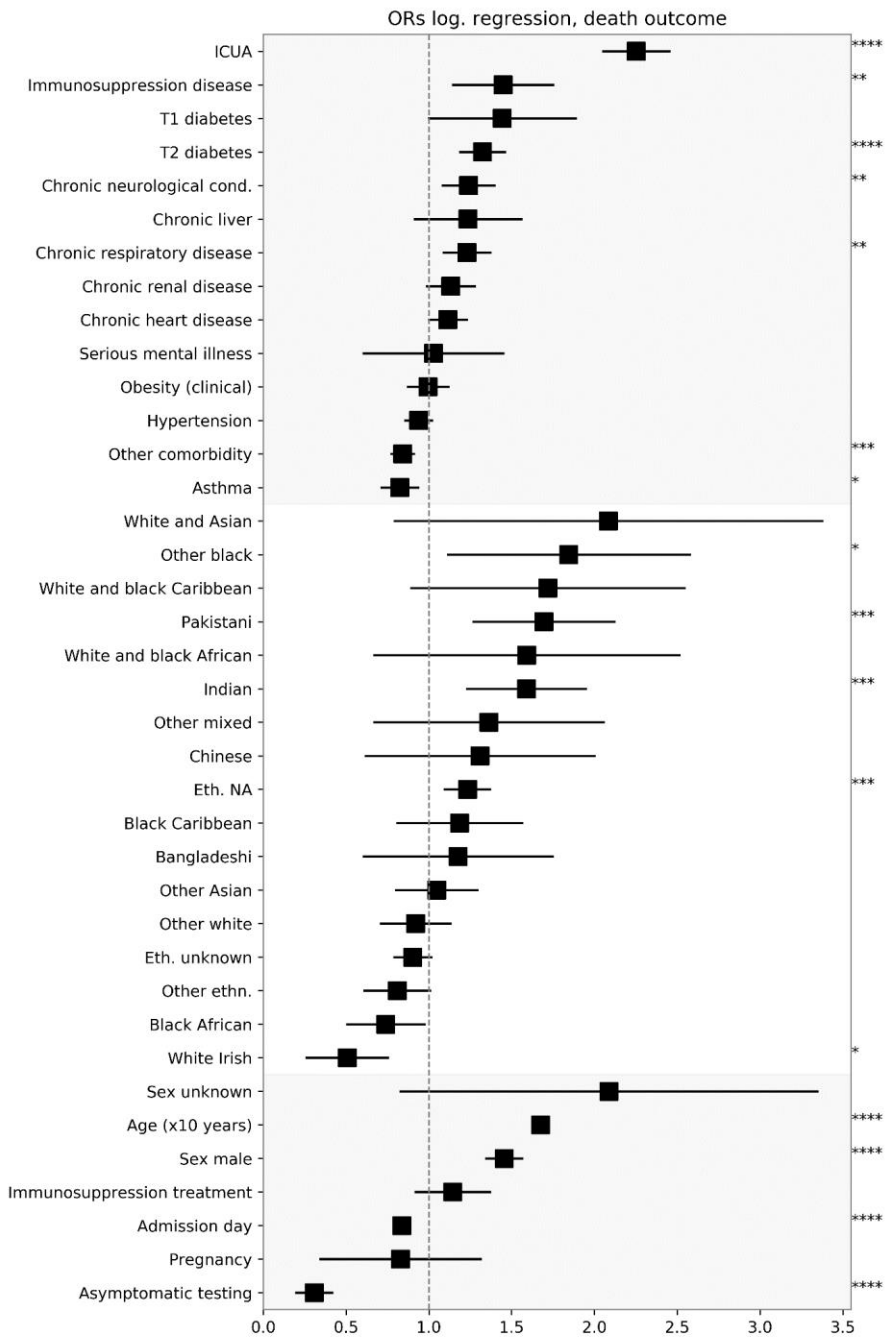

Fig E. Odd ratios (ORs) for death outcome from Table A. Features grouping and stars codes for significance as in Figs A and B.

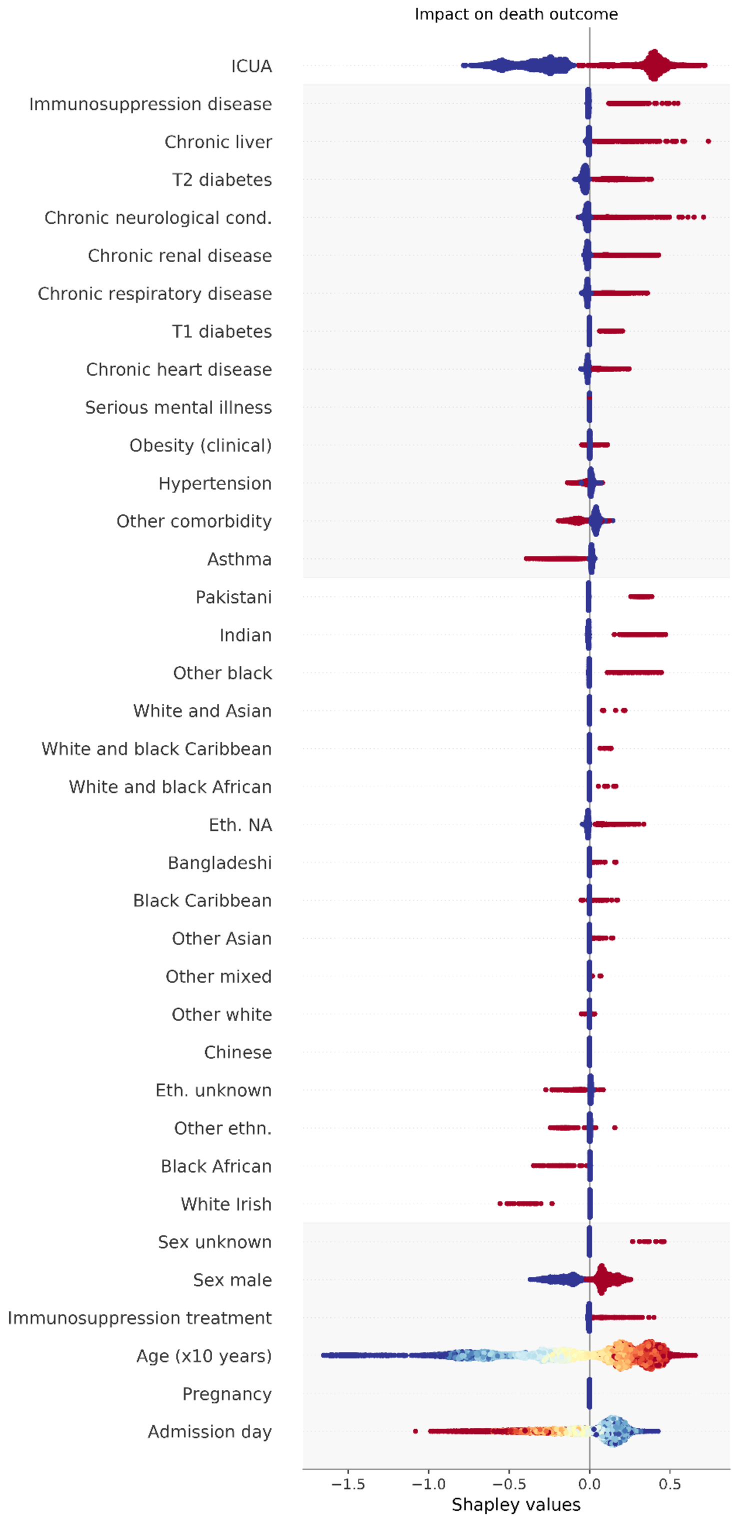

Fig F. Summary plot of Shapley values for impact on death outcome stratifying on ICUA. Colours and keys as in Figs C and D.
