# Supplementary material for: Contrasting factors associated with COVID-19-related ICU admission and death outcomes in hospitalised patients by means of Shapley values
Source: PLoS Comput Biol. 2021 Jun 23;17(6):e1009121. doi: 10.1371/journal.pcbi.1009121 (PMC8259985; doi:10.1371/journal.pcbi.1009121)
Supplement: Attachment — Submitted filename: Reply to reviewers.pdf [file pcbi.1009121.s002.pdf]

Dear Editor,

We would like to thank the two reviewers for carefully studying our manuscript, their overall positive assessment and their constructive comments for improvements and clarity. We have significantly revised and improved our manuscript based on their input as described in the point-by-point response below and implemented in the amended manuscript and by two new supplemental figures and one supplemental table. We also now cite 7 new references ([1-7]) in the revised manuscript)

Reviewer #1

This is a (very) large retrospective examination of the ability of different modeling techniques to predict high-risk COVID patients (patients who died and those who were admitted to the ICU) and to quantify the predictive strength of included predictors. Through their comparison of modeling techniques (logistic vs gradient-boosted tree), they were able to show the difference in predictive ability of machine learning techniques. Further, by examining both the resulting odds ratios and Shapley additive explanations (a novel approach to tool interpretability) they were able to show the relative predictive strength of included factors were similar regardless of model type. This analysis is both topically and methodologically relevant and is a great addition to the existing COVID prediction and overall prediction literature. However, there are some concerns that need to be addressed prior to publication.

Overall:

1.1. The study population was limited to patients who required hospitalizations (stated in the methods). This restriction in the population is not clear in other parts of the analysis (though it is stated in the conclusion) but has a very meaning impact on the generalizability of the study results.

We have amended the manuscript to make it completely clear that the population is patients in hospital. Firstly, we have added "**in hospitalized patients**" to the title, secondly as well as originally identifying the data set as "The COVID-19 Hospitalization in England Surveillance System (CHESS)," we have additionally stated that (added text in bold):

Page 2, line 74 "[…] collects extensive data on patients **admitted to hospital**, including their known comorbidities and important demographic information (such as age, sex, and ethnicity)."

Same page, line 77 "analysis was performed" → "**we performed analyses on this dataset**"

1.2. It is not clear to me if the main purpose of the paper is to compare the predictive modeling approaches and how predictors ranked by model or if it was the comparison of ICU vs Death outcome predictions tools or if predictor importance was the primary goal. I think the abstract and introduction would benefit from a hypothesis type sentence outlining the primary and secondary aims of the work.

It is very much both. We have made this more explicit. Where we had noted that the "objective of this study is two-fold" in the abstract we now make explicit both the substantive and methodological question "The objective of this study is two-fold**, one substantive and one methodological: substantively to […]**" with other edits to reduce the word count making room for this.

We also updated the “Author summary” to introduce the interpretation and predictor ranking bases on the Shapley value analysis:

“**We derived importance scores based on Shapley values which were consistent with the ORs,** […]”.

1.3. What is the vision for the use of the Shapley values or ORs in the clinical decision-making space?

We expanded the “Discussion” section, by explicitly recommending the use of GBDTs over logistic regression to assist clinical decision making (page 10, line 388):

“**Shapley-value analyses allow clinical interpretation of the results from a complex machine-learning model such as the GBDT. Using these we have derived importance scores which are consistent with the better known ORs as an overall assessment of an average effect but can additionally display the extent to which this average effect is consistent across patients or highly variable among different patient groups. We recommend the wider adoption of Shapley-value analyses to support interpretation of ML outputs in clinical decision making given this capacity to communicate the variation in the effects of predictive variables.**”

Methods

2.1. Why was a gradient-boosted tree selected for over an alternative ML approach (random forest, neural net, etc)?

Random forests are in effect similar and could have been chosen while, to the best of our knowledge, neural networks are ideal to tackle unstructured data such as images or sounds. We chose a gradient-boosted tree as one suitable machine learning method. We have made this clear and also that it is used as an exemplar ML method rather than the only possible one to use. Our focus is on the power of any appropriate ML approach combined with a means interpreting and communicating the results. As above (1.3) we have generalised text to consider the use and interpretation of “ML outputs” rather than this being restricted to gradient-boosted decision trees.

We have also added into the methods section on our choice text as below (page 3, line 118).

“In addition, we applied a “gradient boosted decision tree” (GBDT) machine-learning model with logistic objective function**, as an appropriate machine learning approach**.”

2.2. It is great that authors examined the VIF to ensure that collinearity was not a concern during their interpretation of predictor importance -nice job. However, it is not clear to me that the authors accounted for the large number of pvalues they examined. Was any multiple-testing approach applied to the results, if so, which one and why was that the approach selected?

We thank the reviewer for highlighting the importance of accounting for multiple testing.

We reported the p values for all estimated ORs in Table 3. These p values correspond to the null hypothesis that there is no association between the predictor and the outcome; in the original manuscript, we used the standard significance threshold of 0.05 to discriminate significant ($P<0.05$) from non-significant ($P>0.05$) associations. Our main aims were around the overall patterns of prediction, however some readers might well focus on individual factors and our statistical estimates for their significance. When used for this purpose our p values are inadequate. The reviewer correctly argues that this latter step requires accounting for multiple testing. In the revised paper, we applied the Benjamini-Hochberg procedure [3], tested each individual estimate with a false discovery rate of 0.05, and updated the “Author summary”, “Results”, and “Methods” sections accordingly. Five factors

(namely, “obesity” and “white Irish”, “white and black Caribbean”, “black Caribbean”, and “unknown” ethnicities), which previously tested weakly associated with death (0.02<P<0.05), now appear to be non-significant risks for death. This doesn’t alter any of the main conclusions of the work.

2.3. How were comorbidities selected for and what justified specific comorbidities to be examined individually vs. grouped?

We considered all the comorbidities recorded in the CHESS dataset as documented in reference [5]. The comorbidity categories were established and recorded by PHE. We analysed co-morbidities individually because we were interested in whether they would differentially affect ICU admission and death. Grouping them would not allow us to look at this differentiation. To clarify this aspect, we updated the sentence in the “Description of cohort and outcomes” section (page 3 line 96):

“We included all available chronic and pre-existing morbid conditions **recorded by PHE** as potential risk factors […]”,

and also directly cited reference [5] in line 99.

1.4. The employed variable importance approach for the GBDT (Shapley Additive Explanation) is novel and its utility is well outlined. Where other types of variable importance estimation tools (such as partial dependence plots – PDPs) considered in this analysis and why was the Shapley method selected in the end?

We agree that there are other useful approaches for variable importance in ML. We are not claiming to do a comprehensive review of these but to compare one appropriate ML analysis and interpretation with a more classical statistical analysis. We believe that the Shapley method has very solid theoretical ground, as explained in specialised machine learning and game theory literature [2, 8, 9]. To stress this point, we included the following in the revised manuscript (page 4, line 141):

“The model output satisfies $f_i = \sum_{j=0}^{N} \phi_{ij}$ (**which is the local accuracy property**), where ϕ_{i0} is a bias term. **Importantly, it has been mathematically proven that the Shapley allocation is the only possible one that also satisfies two additional desirable properties, i.e., consistency (if a feature’s contribution increases or stays the same regardless of the other inputs, its Shapley value does not decrease), and missingness (a zero-valued feature contributes a zero Shapley value) [2, 8, 9]”**

And in the same page, line 153:

“Such an approach explains each individual prediction f_i and is therefore referred to as a *local* method. In contrast to that, as a complementary *global* method, we consider the so-called partial dependence plots (PDPs) to show the average effect of age and admission date on the predicted outcomes, marginalizing over the values of all other features [1]”.

In addition to this, as suggested by the reviewer, we included the PDPs in Figures 6 and 7 (A-B).

Strikingly, these PDPs mirror the SHAP main effect plots of Figures 6 and 7 (C-D). This is another great result of the Shapley value analyses, which is commented in the “Discussion” section (page 9, line 365):

“**These results mirror the PDPs outlined in Figures 7 and 8 A-B, showing that a local explanation technique such as the Shapley value analysis supersedes and is consistent with the global explanation of the PDPs.**”

Results

3.1. Given the differences in the predictive direction of some of the included features between death and ICU admission, it would be very helpful to have a breakdown of how these to outcomes overlapped – how many patients died within the ICU and what not. Further, if you stratify on ICU admission within the death prediction, is there a difference in predictive validity?

The breakdown of patient outcome is explicitly stated at the beginning of "Methods: description of cohort and outcomes" section (page 3, line 91 of the revised manuscript).

We expect that ICU admission and death are strongly associated. In order to quantify this better, as suggested with the reviewer, we performed both logistic regression and GBDT classification for death outcome including the ICU admission as a predictor. Their performances in predicting death were only marginally better than the models that did not stratify on ICU. The results are illustrated and summarised in the Supplemental Figures S5 and S6, in the Supplemental Table S1, and in the "Results" section (page 6 line 229):

"**Stratifing on ICUA yields marginally higher ROC-AUC scores (logistic regression 0.69 (95% CI 0.66-0.72), GBDT 0.70 (95% CI 0.67-0.72) compared to death prediction obtained without ICUA prediction. In fact, ICUA is a very strong predictor of death (OR 2.25, 95% CI 2.04-2.48) but is markedly correlated to other features (Figure 1). The full results are summarised in Figures S5 and S6, and Table S1.**"

3.2. I am not sure what this sentence means "Generalized collinearity diagnostics by means of variance inflation factor (VIF) excluded severe collinearity (Table 4)." Based on the table heading, it seems like no VIFs were > 2 but that is not clear in the written results.

We accordingly edited this sentence in the main text to: "Generalized collinearity diagnostics by means of variance inflation factor (VIF) excluded severe collinearity **(VIFs<2,** Table 4**, see also reference [4])"**, in order to clarify that VIFs are smaller than a collinearity threshold as recommended in literature.

3.3. For comparing the ORs and Shapley Values, it would be helpful to see of the predictor order differs in a single plot. This is somewhat displayed by Figure 5 but I still found myself looking at the supplement and comparing the predictor order in my head.

We thank the reviewer for this comment. The predictor order estimated by as odds ratios by logistic regression is consistent with that obtained from the Shapley values. In the revised manuscript, we added annotations to Figure 5, thus clearly indicating which predictor each scatter point refers to. In addition to that, Table 3 displays both ORs and Shapley importance scores so that it is possible to appreciate their concordance.

3.4. Since the logistic and the GBDT were similarly predictive, it surprises me that the Shapley values show interaction importance and non-linear associations (something not included in the logistic). This makes me think that examining the Shapley results to this level of graduality may be a reach or that the GBDT ended up over fit despite hyper-parameter selection. Please speak to how these associations can be found meaningful yet a model that does not include them has essentially equal predictive validity.

The performance of the GBDTs (AUC 0.68 (95% CI: 0.66-0.71) and 0.83 (95% CI: 0.81-0.85) for death and ICUA outcome, respectively) were marginally better than the logistic regression (AUC 0.68 (95%

CI 0.65-0.71) and 0.8 (95% CI 0.77-0.82)) for this data set. We argue that that the overall performance in classification of the two models (GBDT and logistic) are similar for this dataset in part because all but two predictors (age and admission date) are binary. Indeed, the logistic model predictions depend on a linear combination of the predictor values, which is adequate if the predictors are binary.

The non-linear associations are important for the age and admission date which are not binary. This is illustrated in Figures 6 and 7 (subfigures C and D) for the death and ICUA prediction, respectively. It is worth noting that the GBDT learns that the impact of the age on ICUA abruptly drops at the 60 years of age. This relation cannot be represented well as a linear function of the age and is captured only by the GDBT (which incidentally has the best performances in ICUA prediction (AUC 0.83).

To address this point in the revised manuscript, we included the following paragraph in the "Discussion" section (page 9 line 367) to make clear that better performance overall is small on the one hand, but to also emphasise the advantages in detecting non-linearity and variation in predictive effect:

"**The performance gains of the GBDTs here are small, in part due to the fact that all but two predictors (age and admission date) are binary. Indeed, the logistic model predictions depend on a linear combination of the predictor values, which is adequate if all the predictors are binary and the classes are linearly separable. The similarity in the predictive power for these specific cases should not shadow the other advantages of the GBDTs (including their greater generality and their ability of detecting non-linearity and variation in predictive effect).**".

As the reviewer suggests, we can exclude overfitting, by means of 5-fold cross-validation over the training set in the hyper-parameter selection.

Discussion:

4.1. This sentence "Chinese ethnicity predicted ICU admission (OR 10.2) most strongly, followed by black Caribbean (OR 5.2)" should incorporate that white British is the baseline. That is stated earlier but as a reader I had to go back and find the reference while ready.

We slightly rephrased this part accordingly (also including consistent rounding, see question 5.2):

"Chinese ethnicity predicted ICU admission (OR 10.2**2 with respect to the white British baseline**) most strongly, followed by black Caribbean (OR 5.2**5**)"

4.2. The fact that the GBDT and the logistic regression had similar predictive validity is not discussed in the discussion. Given that they are similarly predictive, is there a reason to use the GBDT over the logistic regression in this scenario (or vice versa). The authors removed the interpretability issue of GBDT – which is great – so which should I choose?

We updated the "Discussion" section accordingly, where we recommended using GBDT given that interpretability issues can be solved. Please also see our replies to points 1.3 and 3.4.

Minor

5.1. Missing word or typo in the following sentence: "A GBDT aggregates a large number of weak prediction models, in this case decision trees, into a robust prediction algorithm, where the presence of many trees mitigates the errors due a single-tree prediction."

We edited the text accordingly.

5.2. A few typos found in the results – examples below:

a. Space between 95% CI in some and not other results

b. "associated with death" to "associated to death"

c. Inconsistent rounding

We edited accordingly. We thank the referee for bringing these typos to our attention.

5.3. Discuss associations in the same direction (predictor to outcome) through the results. Currently, it varies from sentence to sentence and is hard to follow.

We edited the manuscript accordingly.

Reviewer #2

Overall, I appreciate the size of this study compared to many others in the space with almost 14,000 cases and this seems to meet an important need for addressing the causes of adverse outcome in COViD-19 with sufficient sample size. Also, the approach of feature-wise calculation of odds ratios and assessment of a subsequent model with all features is clearly laid out and easy to follow. The paper agrees with some other published associations which the authors do a good job of describing and I view that these findings and their substantial underlying dataset are important to add to demonstrate such concordance or divergence.

I find the assessment of model performance with Shapley values as well as the calculation of Variance Inflation Factors to assess collinearity to also be a valuable added point of diligence in the analysis. The conclusions drawn seem well supported by the methods used as the authors are not trying to extrapolate mechanistic or far-reaching explanations but are instead trying to clearly demonstrate the associations between various factors and outcome.

I have a few small improvements that I would suggest:

- One very small point, there seems to be two acronyms for the same entity: ICA and ICUA both for Intensive Care Unit Admission. I'm assuming these are meant to be the same thing in my review.

We thank the referee for their positive comments and for bringing this point to our attention. These indeed are both acronyms for the Intensive Care Unit Admission. For consistency, we opted to use only ICUA and revised title, manuscript, and figure labels accordingly.

- Shapley value analysis is a compelling way to present and describe model behavior and the authors do a good job of discussing the implications of this. One thing that would also be interesting to show are the patients for whom the prediction of mortality or ICU admission was most incorrect and which features were present for those patients. This would be a value-added part of discussing predictive model behavior.

We thank the referee for this positive comment and agree that it would be interesting to characterise the group of patients for whom a prediction is incorrect. It is known that these lay along the so called "decision boundary", which identifies, by definition, an ambiguous region of the feature space. We believe that using Shapley value to characterise the decision boundary is an important line of study but that a full treatment of it would add another substantial dimension to a paper where reviewer one

has emphasised that we are already including a lot and must focus on making the existing parts clear to the reader; we have not added this extra dimension to the work but we have now suggested it as a topic for further research in the "Discussion" section (page 10, line 387):

"**Shapley values may also support analytical approaches to address the problem of characterising the group of patients for whom a prediction is incorrect. This is an important additional potential area for research and application.**"

- Figure 2 is particularly interesting and does a good job of summarizing the various relationships between features, death, and ICU admission. The self-defined ethnicities panel in this Figure can be a bit challenging to follow since so many of the labels are located apart from the points to which they correspond although admittedly I don't see a way to improve this. Perhaps some color-coding of the various points and their CI bars would by ethnicity category would help.

In the revised manuscript, the figure has been edited according to the journal guidelines and has higher resolution, which guarantees readability. For this figure, we really recommend not changing the colour coding. There are 19 self-defined ethnicities and we found that implementing a colour map with 19 colours would be more confusing than it is now. Nor do we recommend using few colours for chosen groups of ethnicities. Each point has been linked to its annotation by an arrow, thus permitting the display of all information without any overlap. The numerical data (summary statistics) that underly this graph is also provided in table 3 and so can be used to explore the result.

- One additional small item that I think would be a nice addition is some explanation of how the demographics and deaths datasets were joined together as these cannot be provided by the authors upon publication, someone wishing to recapitulate or extend these findings may benefit from this detail.

We did not need to join datasets together as for each patient the CHESS database provides the quantities used in our study [5]. To avoid confusion, in the revised manuscript we cited reference [2] in the "Description of cohort and outcome" subsection (page 3 line 99) and corrected the data availability statement. As the reviewer notes we are not able to give access to this dataset – but others can also apply to the data controller for access to repeat our analyses.

1. Friedman, J.H., *Greedy function approximation: A gradient boosting machine.* Annals of Statistics, 2001. **29**(5): p. 1189-1232.
2. Shapley, L.S., et al., *A VALUE FOR n-PERSON GAMES*, in *Contributions to the Theory of Games (AM-28), Volume II*, H.W. Kuhn and A.W. Tucker, Editors. 1953, Princeton University Press. p. 307-318.
3. Benjamini, Y. and Y. Hochberg, *Controlling the False Discovery Rate - a Practical and Powerful Approach to Multiple Testing.* Journal of the Royal Statistical Society Series B-Statistical Methodology, 1995. **57**(1): p. 289-300.
4. Fox, J. and S. Weisberg, *An R companion to applied regression*. Third edition / ed. 2019, Los Angeles: SAGE. xxx, 577 pages.
5. *Letter: COVID-19 Hospitalisation in England Surveillance System (CHESS) – daily reporting*. 2020 13 March 2020 [cited 2021 6 April 2021]; Available from: https://www.england.nhs.uk/coronavirus/publication/letter-covid-19-hospitalisation-in-england-surveillance-system-chess-daily-reporting/.
6. Booth, A.L., E. Abels, and P. McCaffrey, *Development of a prognostic model for mortality in COVID-19 infection using machine learning.* Modern Pathology, 2021. **34**(3): p. 522-531.

7. Zoabi, Y., S. Deri-Rozov, and N. Shomron, *Machine learning-based prediction of COVID-19 diagnosis based on symptoms.* Npj Digital Medicine, 2021. **4**(1).
8. Lundberg, S.M., et al., *From local explanations to global understanding with explainable AI for trees.* Nature Machine Intelligence, 2020. **2**(1): p. 56-67.
9. Lundberg, S.M. and S.I. Lee, *A Unified Approach to Interpreting Model Predictions.* Advances in Neural Information Processing Systems 30 (Nips 2017), 2017. **30**.
